# Explainable artificial intelligence for gait analysis: advances, pitfalls, and challenges - a systematic review

**DOI:** 10.3389/fbioe.2025.1671344

**Published:** 2025-10-30

**Authors:** Liangliang Xiang, Zixiang Gao, Peimin Yu, Justin Fernandez, Yaodong Gu, Ruoli Wang, Elena M. Gutierrez-Farewik

**Affiliations:** ^1^ KTH MoveAbility, Department of Engineering Mechanics, KTH Royal Institute of Technology, Stockholm, Sweden; ^2^ Human Performance Laboratory, Faculty of Kinesiology, University of Calgary, Calgary, AB, Canada; ^3^ Faculty of Sports Science, Ningbo University, Ningbo, China; ^4^ Auckland Bioengineering Institute, University of Auckland, Auckland, New Zealand; ^5^ Department of Engineering Science and Biomedical Engineering, University of Auckland, Auckland, New Zealand; ^6^ Department of Women’s and Children’s Health, Karolinska Institute, Stockholm, Sweden

**Keywords:** gait analysis, machine learning, explainable artificial intelligence (XAI), biomechanics, black-box models

## Abstract

Machine learning (ML) has emerged as a powerful tool to analyze gait data, yet the “black-box” nature of many ML models hinders their clinical application. Explainable artificial intelligence (XAI) promises to enhance the interpretability and transparency of ML models, making them more suitable for clinical decision-making. This systematic review, registered on PROSPERO (CRD42024622752), assessed the application of XAI in gait analysis by examining its methods, performance, and potential for clinical utility. A comprehensive search across four electronic databases yielded 3676 unique records, of which 31 studies met inclusion criteria. These studies were categorized into model-agnostic (n = 16), model-specific (n = 12), and hybrid (n = 3) interpretability approaches. Most applied local interpretation methods such as SHAP and LIME, while others used Grad-CAM, attention mechanisms, and Layer-wise Relevance Propagation. Clinical populations studied included Parkinson’s disease, stroke, sarcopenia, cerebral palsy, and musculoskeletal disorders. Reported outcomes highlighted biomechanically relevant features such as stride length and joint angles as key discriminators of pathological gait. Overall, the findings demonstrate that XAI can bridge the gap between predictive performance and interpretability, but significant challenges remain in standardization, validation, and balancing accuracy with transparency. Future research should refine XAI frameworks and assess their real-world clinical applicability across diverse gait disorders.

## 1 Introduction

Gait is a complex motor activity governed by neuromuscular coordination and biomechanics, and it serves as a key indicator of an individual’s overall health status. In clinical practice, gait analysis is widely used to detect pathological changes such as freezing of gait in Parkinson’s disease, post-stroke hemiparetic gait, or compensatory patterns following musculoskeletal injury (Filtjens et al., 2021; Apostolidis et al., 2023). For example, approximately 60%–80% of stroke survivors experience gait impairments (Cirstea, 2020), and more than 80% of individuals with Parkinson’s disease develop gait disturbances during the disease course (Faerman et al., 2025). Thus, gait analysis has become a critical tool across clinical, sports, and rehabilitation contexts, enabling the assessment of movement patterns and functional impairments (Winter, 2009). As populations age and mobility-related issues become more prevalent, the need for accurate and comprehensive gait evaluation methods continues to grow.

Marker-based motion capture systems have been the gold standard in gait analysis for decades, offering comprehensive tracking of whole-body kinematics with high temporal and spatial resolution. In addition to these systems, gait analysis has employed various technologies such as electromyography, pressure mats, wearable sensors, and bi-planar fluoroscopy. While bi-planar fluoroscopy provides high-precision tracking of skeletal motion, its application is limited to small capture volumes and specific anatomical regions. Moreover, the high cost, limited accessibility, and radiation exposure associated with fluoroscopy restrict its suitability for routine clinical use (Kessler et al., 2019).

Despite their accuracy, optical marker-based systems are typically limited to controlled laboratory environments, reducing their feasibility for broader, real-world assessments (Chen et al., 2016). Recently, markerless motion capture technology has emerged as a promising alternative, using computer vision algorithms to track body movements without physical markers (Wade et al., 2022; Uhlrich et al., 2023). While markerless systems avoid issues related to marker placement, they often struggle with accurate pelvis tracking and their performance depends heavily on the training data, limiting applicability to populations with atypical gait, such as prosthesis users. Wearable sensors, such as accelerometers and gyroscopes, enable data collection in real-world settings but face challenges including signal drift, calibration errors, and soft tissue artifacts, which can compromise accuracy (Chen et al., 2016; Xiang et al., 2022b; Xiang et al., 2023). While these technologies have advanced the field considerably, each has limitations in accuracy, accessibility, and cost, which constrain their widespread use and the ability to comprehensively understand complex gait patterns.

Machine learning (ML) has become a powerful and transformative tool in biomechanics to address some of the limitations in traditional gait analysis methods (Halilaj et al., 2018; Xiang et al., 2022b; Xiang et al., 2025). ML algorithms can process large, high-dimensional datasets to extract meaningful features, enabling accurate classification of walking patterns, detection of gait abnormalities, and prediction of joint mechanics and clinical outcomes (Ferber et al., 2016; Halilaj et al., 2018; Phinyomark et al., 2018; Harris et al., 2022; Xiang et al., 2022a; 2023; 2024; Gao et al., 2023; Mekni et al., 2025a; Mekni et al., 20252025b). By automating the analysis process, ML reduces reliance on manual interpretation, offering a more efficient and scalable approach. However, these advancements come with challenges, primarily concerning the ‘black-box’ nature of many ML models, which prioritize predictive accuracy at the expense of transparency, rather than the inherently interpretable ‘clear-box’ models, such as linear regression or simple decision trees. This lack of interpretability raises concerns about their reliability, particularly in clinical contexts where transparency is critical for informed decision-making (Adadi and Berrada, 2018; Salahuddin et al., 2022; Yang et al., 2022; Albahri et al., 2023; Frasca et al., 2024).

Explainable Artificial Intelligence (XAI) has the potential to bridge the interpretability gap by providing insights into how and why ML models make specific predictions. In healthcare, XAI has become a prominent tool, particularly for enhancing the trustworthiness and explainability of AI-driven outcomes (Adadi and Berrada, 2018; Tjoa and Guan, 2020; Vilone and Longo, 2021; Loh et al., 2022; Minh et al., 2022; Salahuddin et al., 2022; Yang et al., 2022; Albahri et al., 2023), though its application in gait analysis remains relatively new. However, concerns have been raised that *post hoc* explanations may sometimes be misleading or provide only superficial insights, risking bias or false reassurance if not carefully validated (Ghassemi et al., 2021). Addressing these concerns, XAI techniques have the potential to identify which features most influence model outputs, thereby supporting clinicians and researchers in interpreting gait patterns and making more informed decisions (Harris et al., 2022). By improving model transparency, XAI not only fosters trust but also facilitates the discovery of novel biomechanical insights.

Techniques such as Local Interpretable Model-agnostic Explanations (LIME) (Ribeiro et al., 2016) and Shapley Additive Explanations (SHAP) (Lundberg and Lee, 2017) can be applied to a wide range of machine learning models to identify influential input features. This flexibility is particularly valuable in gait analysis, where such methods can reveal biomechanical patterns underlying model predictions and enhance the interpretability of complex algorithms (Dindorf et al., 2020; Kim et al., 2022; Teoh et al., 2024). Apart from feature importance, attention maps applied to time-series data also show promise in providing insights by highlighting significant time points or features during the processing of sequential information within neural networks (Xiang et al., 2024). Other XAI methods, such as Gradient-weighted Class Activation Mapping (Grad-CAM) (Selvaraju et al., 2017), have proven useful in highlighting the regions of gait video data that contribute most to model predictions, offering visual explanations that can be especially informative for clinicians (Slijepcevic et al., 2023; Martínez-Pascual et al., 2024; Teoh et al., 2024). Counterfactual explanations can also be applied to demonstrate how small changes in gait characteristics, such as joint angles or stride length, could affect model outputs, allowing for a deeper understanding of decision boundaries in gait anomaly detection (Dou et al., 2023). Although these methods have shown promise, their application in gait analysis remains limited, indicating a need for further exploration into which techniques best suit the specific demands of gait-related data and tasks.

Despite recent advancements, significant gaps remain in the application of XAI to gait analysis. Current ML applications in gait analysis emphasize predictive accuracy over interpretability, creating a gap that limits their clinical utility (Harris et al., 2022; Frasca et al., 2024). This lack of transparency can hinder the adoption of ML in clinical gait analysis, reducing its potential impact. Thus, there is a need for a comprehensive review of XAI approaches in gait analysis to assess their current capabilities, limitations, and areas for future improvement. By examining the intersection of XAI and gait analysis, this review aims to highlight the opportunities for advancing this field and uncover the potential of XAI to enhance decision-making in gait-related research and clinical practice.

## 2 Methods

The protocol for this systematic review was designed following the Preferred Reporting Items for Systematic Reviews and Meta-Analyses (PRISMA) guidelines (Moher et al., 2009) to ensure methodological rigor and transparency. Additionally, the review protocol was registered with the International Prospective Register of Systematic Reviews (PROSPERO) (CRD42024622752).

### 2.1 Search strategy

A systematic search was conducted across four electronic databases—Scopus, Web of Science, IEEE Xplore, and PubMed—covering the period from January 2000 to October 2024. The search employed keywords combined with Boolean operators, as outlined in Table 1. To ensure comprehensive coverage, the bibliographies of relevant academic articles were also reviewed for additional studies. Titles, abstracts, and full texts of the retrieved records were carefully screened to assess their relevance.

**TABLE 1:** Boolean search strings employed for the corresponding bibliographic databases and search engines.

Database	Boolean search strings	Limit conditions
Scopes, Web of Science, IEEE Explore	(“gait” OR “walk*” OR “locomotion” OR “ambulation” OR “movement”) AND (“XAI” OR “explainab*” OR “interpret*” OR “SHAP” OR “LIME” OR “class activation mapping” OR “Grad-CAM” OR “DeepLIFT” OR “LRP” OR “saliency map*” OR “attention map*” OR “feature importance” OR “counterfactual explanation”) AND (“AI” OR “artificial intelligence” OR “machine learning” OR “deep learning” OR “neural network*” OR “CNN” OR “RNN” OR “LSTM” OR “attention mechanism” OR “SVM” OR “KNN” OR “decision tree” OR “ensemble learning” OR “*boost*” OR “random forest” OR “naive bayes”)	Keywords in all field of the article; Advanced search; Article type: Journal; Language: English; Publish time: From 2000 to October 2024
PubMed	[(“gait” OR “walk*” OR “locomotion” OR “ambulation” OR “movement”) AND (“XAI” OR “explainab*” OR “interpret*” OR “SHAP” OR “LIME” OR “class activation mapping” OR “Grad-CAM” OR “DeepLIFT” OR “LRP” OR “saliency map*” OR “attention map*” OR “feature importance” OR “counterfactual explanation”) AND (“machine learning” OR “deep learning” OR “artificial intelligence” OR “neural network*” OR “AI” OR “SVM” OR “random forest” OR “decision tree”)]	Keywords in all field of the article; Advanced search; Article type: Journal; Language: English; Publish time: From 2000 to October 2024

XAI, explainable artificial intelligence; LIME, Local Interpretable Model-agnostic Explanations; SHAP, shapley additive explanations; Grad-CAM, Gradient-Based Class Activation Mapping; DeepLIFT, Deep Learning Important FeaTures; LRP, Layer-Wise Relevance Propagation; CNN, convolutional neural network; RNN, recurrent neural network; LSTM, Long Short-Term Memory; SVM, support vector machine; KNN, K-Nearest Neighbors.

### 2.2 Eligibility criteria

Eligibility criteria were defined based on the Participants, Intervention, Comparisons, and Outcomes (PICO) framework to ensure systematic and consistent data extraction. This extraction focused on population characteristics (e.g., sample size, gender, age, health condition), gait analysis method, ML or deep learning model used, explainable methods (e.g., SHAP, LIME), ML task (e.g., classification, regression). outcomes measured, performance metrics.

The selection process was conducted independently by two reviewers (L.X. and Z.G.). Disagreements regarding study inclusion were resolved through discussion, and if consensus could not be reached, a third reviewer (J.F.) made the final decision. Studies were excluded based on the following criteria: (1) Studies that did not incorporate any explainability methods (e.g., SHAP, LIME) applied to black-box models, or that relied solely on inherently interpretable models (e.g., linear regression) without additional XAI components; (2) Focused exclusively on animal gait or used animal models without relevance to human gait analysis; (3) Lacked methodological details or experimental data; (4) Did not use quantitative measures to evaluate gait characteristics or relied solely on qualitative approaches without empirical analysis; and (5) Studies were excluded if the predictive models did not report performance metrics or failed to achieve a baseline level of validity, since explanations derived from poorly performing models would not provide meaningful or reliable insights into gait biomechanics. Search results from each database were imported into EndNote X9 (Thomson Reuters, California, USA) to manage references and streamline the screening process.

### 2.3 Risk of bias assessment

The risk of bias was assessed using a modified Downs and Black checklist (Downs and Black, 1998) adapted for sports science, healthcare, and rehabilitation studies and supplemented with XAI-specific items (Table 2). Two reviewers (L.X. and Z.G.) independently evaluated study quality, achieving >85% initial agreement. Given the small number of included studies, a formal inter-rater statistic (e.g., Cohen’s κ) was not calculated. The evaluation consisted of 12 distinct criteria, each rated on a scale of 0 (no), 1 (maybe), or 2 (yes), with a cumulative score ranging from 0 to 24. To ensure objectivity and standardization in quality assessment, the scores were converted to a percentage scale ranging from 0% to 100%.

**TABLE 2:** Risk of bias assessment components.

Section	Number	Description
Inclusion criteria stated	Q1	Inclusion criteria for subjects are clearly stated
Appropriate assignment of subjects	Q2	Subject assignment was appropriate (random/equal baseline)
Description of intervention	Q3	Detailed description of interventions applied
Dependent variables	Q4	Dependent variables defined adequately
Assessment practicality	Q5	Practicality and reliability of assessment methods
Training duration	Q6	Duration of training described (acute vs. long-term)
Statistical analysis	Q7	Appropriate statistical methods used (variability, repeated measures)
Detailed results	Q8	Results presented in sufficient detail (e.g., mean, SD, effect size)
Conclusions	Q9	Insightful conclusions, clear and concise, future directions suggested
ML interpretability	Q10	Interpretability Method: Described methods for explainability (e.g., SHAP values)
	Q11	Validation of Interpretability: Reliability of validation methods Transparency of Predictors: All predictors clearly reported
	Q12	Model Performance: Clearly reported measures (accuracy, F1 score, etc.)

ML, Machine learning; SD, Standard deviation; SHAP, Shapley additive explanations.

## 3 Results

### 3.1 Search results

**FIGURE 1:**
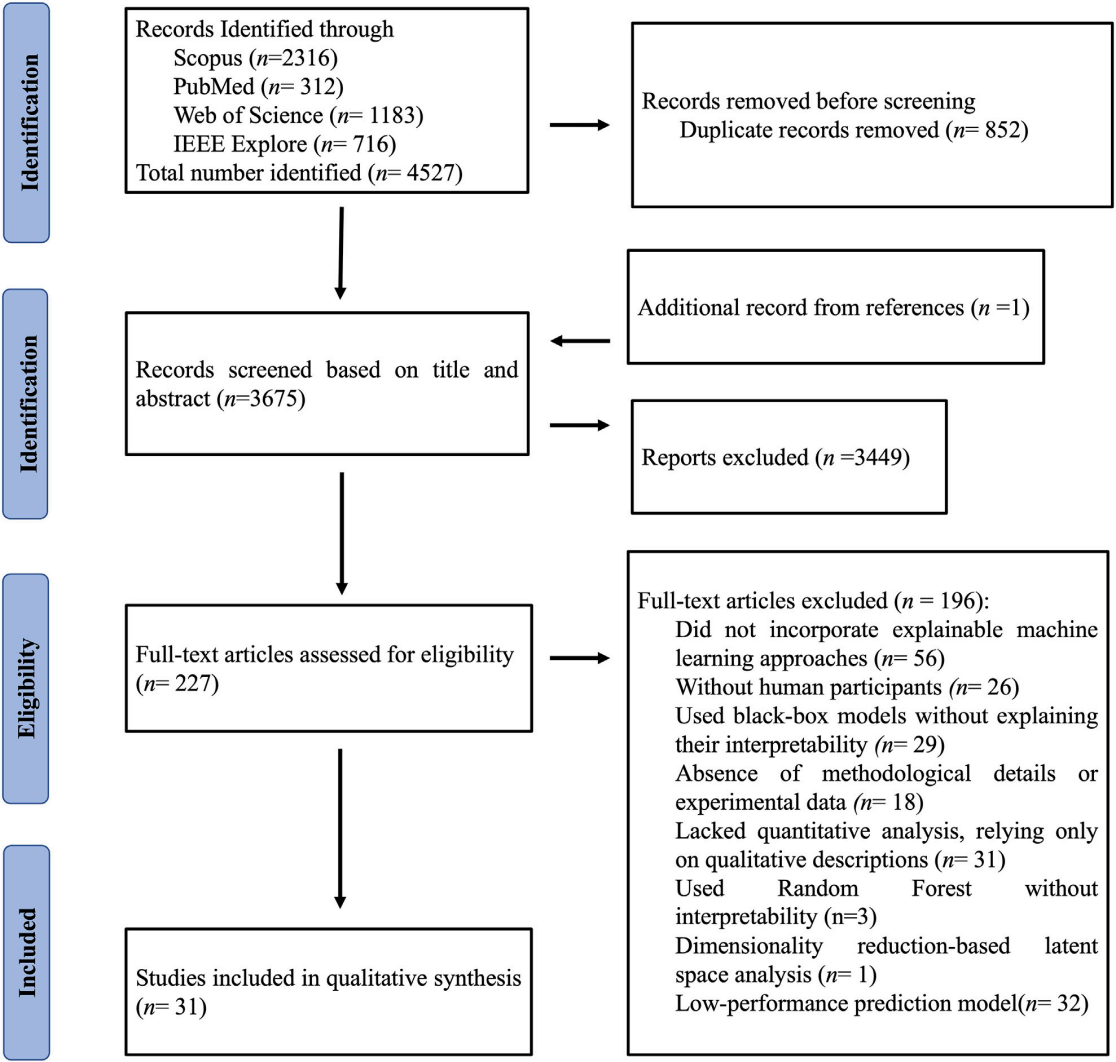
The Preferred Reporting Items for Systematic Reviews and Meta-Analysis (PRISMA) flow diagram of the study selection process.

After removing 852 duplicate records, 3676 unique records were retained for further screening (Figure 1). During the initial screening phase, 3449 records were excluded for being irrelevant or failing to meet the inclusion criteria. Of these, 196 studies were excluded for specific reasons: lack of XAI approaches (n = 56), absence of human participants (n = 26), reliance on black-box models without interpretability (n = 29), insufficient methodological details or experimental data (n = 18), lack of quantitative analysis (n = 31), use of inherently interpretable models rather than *post hoc* explainability methods (n = 3), dimensionality-reduction–based latent space analysis without interpretability (n = 1), and low-performance predictive models (n = 32). Ultimately, 31 studies were included in the qualitative synthesis.

### 3.2 Quality assessment

As summarized in supplementary data (Supplementary Table S1), the quality assessment scores of the 31 included studies ranged from 83.3% to 91.7%, with an average score of 88.7%. All studies followed standardized experimental design protocols, with an average methodology score of 1.82, and provided clear explanations of XAI techniques, achieving an average score of 1.58.

### 3.3 Study characteristics

**FIGURE 2:**
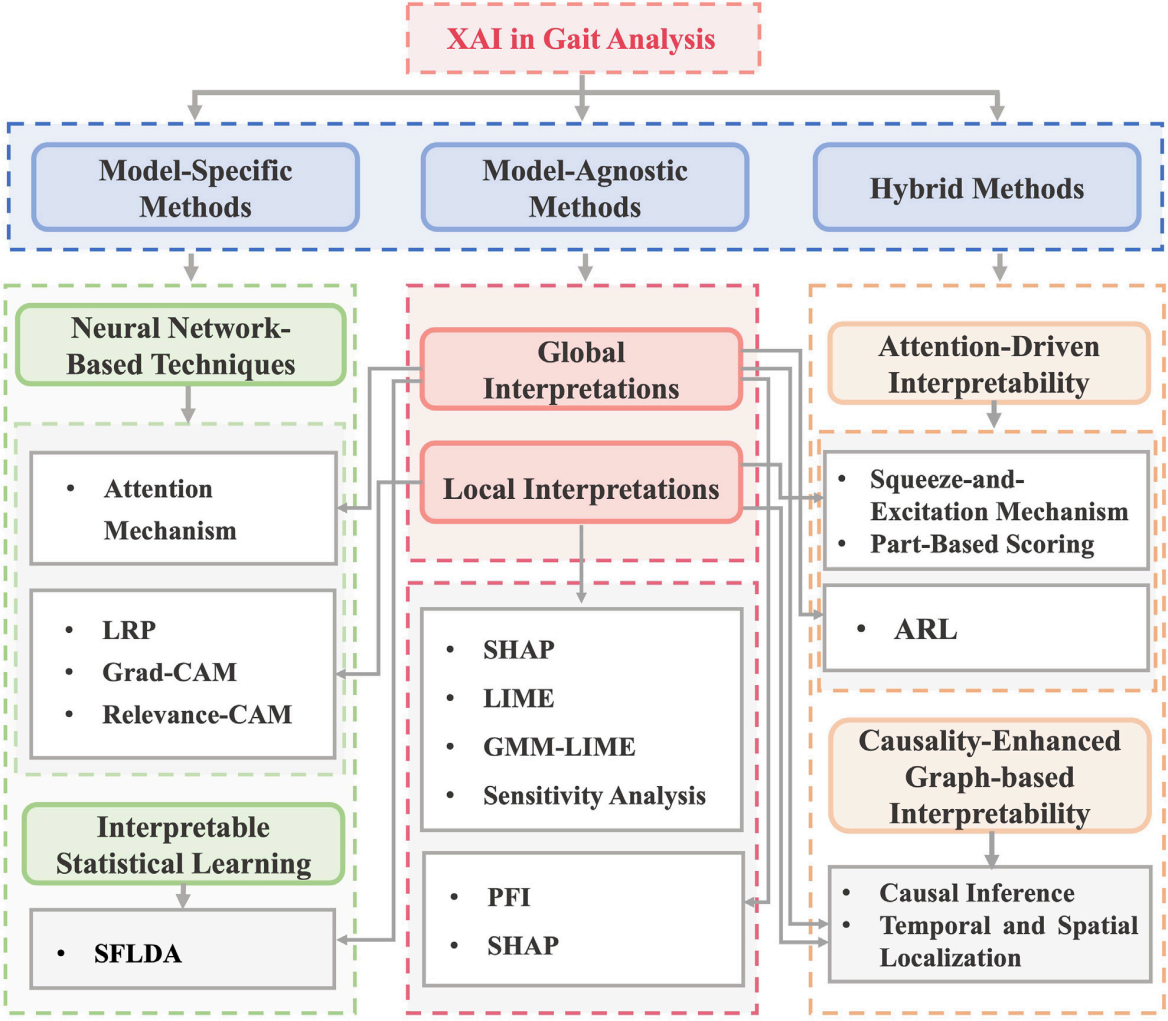
Types of interpretable machine learning models used in the included studies. Note: XAI: Explainable Artificial Intelligence, LRP: Layer-Wise Relevance Propagation, Grad-CAM: Gradient-Based Class Activation Mapping, SFLDA: Sparse Functional Linear Discriminant Analysis, LIME: Local Interpretable Model-agnostic Explanations, SHAP: Shapley Additive Explanations, GMM-LIME: Gaussian Mixture Model - Local Interpretable Model-Agnostic Explanations, PFI: Permutation Feature Importance, ARL: Attention Reinforcement Learning.

The included studies were categorized into three primary methodological types of ML interpretability within gait analysis (Figure 2): model-agnostic (n = 16), model-specific (n = 12), and hybrid (n = 3).

#### 3.3.1 Model-agnostic methods

A total 16 reviewed studies applied ML and XAI within gait analysis across diverse clinical and healthy populations (Dindorf et al., 2020; Davis et al., 2021; Kim et al., 2021; Kim et al., 2022; Rupprechter et al., 2021; Teufl et al., 2021; Kokkotis et al., 2022; Moon et al., 2022; Fan et al., 2023; Zheng et al., 2025; Aghababa and Andrysek, 2024; Hussain and Jany, 2024; Mulwa et al., 2024; Özateş et al., 2024; Trabassi et al., 2024; Wu et al., 2024) (Table 3). Among these, 14 studies employed local interpretation methods to explain instance-specific predictions, while two studies (Teufl et al., 2021; Trabassi et al., 2024) utilized global interpretation techniques to assess overall model behavior. Only one study reported the fidelity of local model explanations (Mulwa et al., 2024).

**TABLE 3:** Model-agnostic interpretability methods in gait analysis using machine learning.

Studies	Participant characteristics: number (age, male\female)	Analysis approach	Machine learning algorithm	Explainable approach	Machine learning type	Data type	Interpretability-related metrics
Rupprechter et al. (2021)	729 (PD, N/A, N/A)	Computer Vision	RF, LDA, LG, SVM, GBT.	SHAP	Classification	Tabular data (GF)	Shapley values
Kim et al. (2021)	20 (10 with sarcopenia, 71.1 years, 10/0; 10 HP, 69.6 years, 10/0)	Mocap	SVM, RF, MLP, CNN, BiLSTM	SHAP	Classification	Time-series (IMUs), Tabular data (spatiotemporal parameters, DSP)	Shapley values
Aghababa and Andrysek (2024)	21 (unilateral transfemoral amputations patients, N/A, N/A)	Mocap	LR, RF, SVM, LightGBM	SHAP	Classification	Tabular data (GF)	Shapley values
Kokkotis et al. (2022)	151 (44 ACLD:18–50 years,31/13; 54 ACLR: 18–50 years, 40/14; 53; HP: 18–50 years, 34/19)	Mocap	SVM, ANN, RF, DT, XGBoost, KNN, LR	SHAP	Classification	Time-series joint angles and GRFs	Shapley values
Wu et al. (2024)	96 (96 PD: 62 years, 41/55)	Mocap	Adaboost, LogitBoost, XGBoost, LR, RF, SVM, KNN, DT, NB, GBM, MLP	SHAP	Classification	Time-series IMU and Tabular data (GF)	Shapley values
Davis et al. (2021)	3925 (2156 female, 64.8 years; 1769 male, 64.1 years)	Mocap	HGBR	SHAP	Regression	Tabular data (gait speed)	Shapley values
Fan et al. (2023)	214 older adults (60–95 years, 58/156)	Mocap	RF, DT, NB	SHAP	Classification	Tabular data (GF, demographic characteristics, and clinical measures)	Shapley values
Zheng et al. (2025)	244 (129 adults, 38.3 years, N/A; 115 elderly, 76.7 years, N/A)	IMU Sensors	CNN, GRU	SHAP	Classification	Signal (IMUs)	Shapley values
Hussain and Jany (2024)	123 (48 stroke patients: 70.6 years, 30/18; 75 HP: 76.3 years, 23/52)	EMG	Gboost,RF, HistGBoost	SHAP, LIME	Classification	Tabular data (EMG Spectral Features)	Shapley values, feature importance
Mulwa et al. (2024)	2313 (2084 MSI patients, N/A, N/A; 229 HP, N/A, N/A)	IMU Sensors; GRF	RF, MLP	GMM-LIME	Classification	Time-series (IMUs, GRFs)	Feature importance
Özateş et al. (2024)	348 (248 various foot conditions:18–82 years, N/A; 100 HP,20–75 years, N/A)	Mocap	SVM, RF, KNN, LR	Lime	Classification	Time-series (joint angles); Tabular data (GF)	Feature importance
Dindorf et al. (2020)	47 (27 HP: 24.63 years, 13/14; 20 THAP: 57.79 years, 7/13)	Mocap	RF, SVM, MLP	Lime	Classification	Tabular data (discrete statistical features from IMUs)	Feature importance
Kim et al. (2022)	42 (21 Osteopenia and/or Sarcopenia patients, 70.4 years, 0/21, 21 controls: 70.33 years, 0/21)	IMU Sensors	XGBoost, RF, SVM, CNN, BiLSTM, ResNet	SHAP, Grad-CAM, Relevance-CAM	Classification	IMU time-series, Tabular data (GF and DSP)	Shapley values, Relevance scores, Gradient-weighted activation map, relevance-weighted activation map
Moon et al. (2022)	40 (HP, N/A, N/A)	Plantar pressure; IMU Sensors	EDN with 1d_CNN layers	Sensitivity Analysis	Recognition	Time-series data (plantar pressure, IMUs)	Relevance scores, sensitivity
Teufl et al. (2021)	46 (25 HP, 24 years, N/A; 20 THAP: 56.9 years, 7/13; 1 PP, 59 years, 1/0)	Mocap	SVM	PFI	Classification	Time-series data (joint angles)	Feature importance
Trabassi et al. (2024)	130 (30 CA, 51.6 years, 17/13; 100 HP, 57.1 years, 40/60)	IMU Sensors	RF, GAN, ctGAN	SHAP	Classification	Tabular data (gait features)	Shapley values

XAI, explainable artificial intelligence; Mocap, Motion Capture; HP, healthy participants, AIS, adolescent idiopathic scoliosis, PD, Parkinson’s Disease, ACLD, Anterior Cruciate Ligament-deficient Prior to Surgery, ACLR, anterior cruciate ligament reconstruciton, ANN, artificial neural networks, AUC, Area Under the ROC, curve, BiLSTM, Bidirectional Long Short-Term Memory, DSP, descriptive statistical parameters, GF, gait features, DT, decision tree, SHAP, shapley additive explanations, GBM, gradient boosting machine, GBT, gradient boosted trees, GMM-LIME, Gaussian Mixture Model - Local Interpretable Model-Agnostic Explanations, Grad-CAM, Gradient-Based Class Activation Mapping, HGBR, histogram gradient boosting regression, HistGBoost, Histogram Gradient Boosting, IMU, inertial measurement units, LDA, linear discriminant analysis, LG, logistic regression, LIME, Local Interpretable Model-Agnostic Explanation, LR, logistic regression, LRP, Layer-Wise Relevance Propagation, LightGBM, light gradient boosting machine, MLP, Multi-Layer Perceptron, MSI, musculoskeletal impairments, NB, Naïve Bayes, SVM, support vector machine, PFI, permutation feature importance, PP, prosthesis participant, RF, random forest, ResNet, Residual Neural Network, SNN, Self-Normalizing Neural Networks, SiNN, siamese neural networks, TNR, true negative rate, TPR, true positive rate, AUROC, area under the receiver operating characteristic curve, CNN, convolutional neural network, GRU, gated recurrent unit, EMG, electromyography, GRFs, Ground Reaction Forces, KNN, K-Nearest Neighbors, EDN, Encoder-Decoder network, CA, cerebellar ataxia, GAN, generative adversarial network, ctGAN, Conditional Tabular GAN.

Local methods, such as SHAP and LIME, dominated the field, with SHAP applied in 11 studies to generate Shapley values for detailed insights into feature contributions (Davis et al., 2021; Kim et al., 2021; Kim et al., 2022; Rupprechter et al., 2021; Kokkotis et al., 2022; Fan et al., 2023; Zheng et al., 2025; Aghababa and Andrysek, 2024; Hussain and Jany, 2024; Trabassi et al., 2024; Wu et al., 2024). SHAP identified vertical ground reaction forces (GRF) and stride duration as key features for classifying Parkinson’s disease (PD) (Rupprechter et al., 2021) and sarcopenia (Kim et al., 2021). Similarly, LIME provided local feature importance scores for tabular and signal data in four studies, such as identifying functional ankle angles in foot pathologies (Özateş et al., 2024).

In contrast, global interpretation methods focused on dataset-wide feature importance. Teufl et al. (2021) applied permutation feature importance (PFI) to rank joint angles and range of motion (ROM) metrics for gait abnormality classification in hip arthroplasty patients, identifying pelvic tilt as the most influential feature. Trabassi et al. (2024) employed SHAP in a global context, aggregating Shapley values across cerebellar ataxia and healthy cohorts to reveal gait symmetry and cadence as key discriminators. These global approaches prioritized population-level insights, with PFI emphasizing feature robustness and SHAP aggregations highlighting biomechanical patterns.

The studies employed diverse data types and algorithms. Motion capture (Mocap) systems was the primary data acquisition method in the majority of studies (Dindorf et al., 2020; Davis et al., 2021; Kim et al., 2021; Teufl et al., 2021; Kokkotis et al., 2022; Fan et al., 2023; Aghababa and Andrysek, 2024; Mulwa et al., 2024; Özateş et al., 2024; Wu et al., 2024), providing time-series joint kinematics, GRF, and functional movement metrics. Wearable sensors (IMUs, EMG) enabled portable signal collection in five studies (Kim et al., 2022; Moon et al., 2022; Zheng et al., 2025; Hussain and Jany, 2024; Trabassi et al., 2024). Tabular gait parameters such as stride duration and cadence were frequently combined with signal data to enhance model performance. In terms of algorithms, ensemble methods (Random Forest, XGBoost) and neural networks [convolutional neural network (CNN), recurrent neural network (RNN)] were preferred for their robustness in handling heterogeneous data. Zheng et al. (2025) used CNNs to classify IMU-derived gait patterns in aging individuals, reporting an accuracy of 81.4% supported by SHAP-based local explanations to elucidate feature influence.

#### 3.3.2 Model-specific methods

Model-specific interpretability techniques were applied in 12 studies focused on gait analysis (Horst et al., 2019; Horst et al., 2020; Aeles et al., 2021; Creagh et al., 2021; Filtjens et al., 2021; Slijepcevic et al., 2021; Slijepcevic et al., 2023; Apostolidis et al., 2023; Yoon et al., 2023; Alharthi, 2024; Kim et al., 2024; Xiang et al., 2024), and were classified into two main categories: (1) neural network-based methods, including Layer-wise Relevance Propagation (LRP), Grad-CAM, and attention mechanisms) and (2) interpretable statistical learning approaches including sparse functional linear discriminant analysis (SFLDA) (Table 4). These approaches were applied across diverse populations, including PD, cerebral palsy (CP), stroke survivors, and healthy participants, to elucidate model decisions and link them to biomechanically relevant features.

**TABLE 4:** Model-specific interpretability techniques in machine learning for gait analysis.

Studies	Participant characteristic: number (group: age, male\female)	Gait analysis method	Machine learning algorithm	Explainable approach	Problem type	Data type	Interpretability-related metrics
Horst et al. (2019)	57 (HP: 23.1, 28/29)	Force plate, Mocap	DNN	LRP	Classification	GRFs, Joint angle	Relevance scores
Alharthi (2024)	166 (93 PD: 66.3, 59/34; 73 HP, 66.3, 40/33)	Force platform	CNN	LRP	Classification	GRFs	Relevance scores
Slijepcevic et al. (2021)	194 disorders patients (37 hip: 44.2, 31/6; 52 knee: 43.5, 37/15; 43 ankle: 42.6, 36/7; 62 HP: 36, 28/34)	Mocap	CNN	LRP	Classification	GRFs	Relevance Scores
Horst et al. (2020)	62 (HP: 23.1, 28/34)	Force platform	CNN	LRP	Classification	GRFs	Relevance scores
Filtjens et al. (2021)	42 (14 PD with FOG: 69.6, N/A; 14 PD without FOG, 66.7, N/A; 14 HP, 65.2, N/A)	Mocap	CNN	LRP	Classification	Joint Angles	Relevance scores
Aeles et al. (2021)	80 (HP: 23.6, 62/18)	Mocap	CNN	LRP	Classification	EMG	Relevance scores
Creagh et al. (2021)	93 (24 HP: 35.6, 18/6; 52 mild MS: 39.3, 16/36; 21 moderate MS: 40.5, 7/14)	IMU Sensors	CNN	LRP	Classification	IMU	Relevance scores
Xiang et al. (2024)	25 (HP: 25.8, 25/0)	Mocap	LSTM-MLP	Attention Mechanism	Regression	Joint angles, torques, contact forces, IMUs	Attention weights
Kim et al. (2024)	38 (AIS adolescents: N/A, 14/24)	IMU Sensors	SVM, RF, CNN	Grad-CAM	Classification	Joint angles	Gradient-weighted activation map
Apostolidis et al. (2023)	64 (34 chronic stroke survivors, >55 years, N/A; 30 HP > 55 years, N/A)	Mocap	SiNN, RF, SVM, NN	Grad-CAM	Classification	Time-series data (joint angle, GRFs); Tabular data (gait features)	Gradient-weighted activation map
Slijepcevic et al. (2023)	302 (302 CP, N/A, N/A)	Mocap	RF, CNN, SNN, DT	Grad-CAM	Classification	Kinematics and GRF	Gradient-weighted activation map
Yoon et al. (2023)	833 (333 with CP, 5–65 years, N/A; 500 controls, 13–76 years, N/A)	Mocap	SFLDA	Sparse discriminative features	Classification	Time-series joint angles	Sparse discriminative feature importance

XAI, explainable artificial intelligence; HP, healthy participants; Mocap, Motion Capture; CNN, convolutional neural networks, IMU, inertial measurement units, LRP, Layer-Wise Relevance Propagation, MLP, Multi-Layer Perceptron, PD, Parkinson’s Disease, RMSE, root mean squared error, SVM, support vector machine, TL, transfer learning, DNN, deep neural network, EMG, electromyography, FOG, freezing of gait, GRFs, Ground Reaction Forces, ALS, adolescent idiopathic scoliosis, MS, multiple sclerosis, CP, cerebral palsy, Grad-CAM, Gradient-Based Class Activation Mapping, SFLDA, sparse functional linear discriminant analysis.

##### 3.3.2.1 Neural network-based interpretability techniques

LRP was the most frequently employed method (Horst et al., 2019; Horst et al., 2020; Aeles et al., 2021; Creagh et al., 2021; Filtjens et al., 2021; Slijepcevic et al., 2021; Alharthi, 2024), applied to CNNs and deep neural networks (DNNs) to explain classifications of gait abnormalities. Horst et al. (2019), Horst et al. (2020) and Alharthi (2024) used LRP to compute relevance scores for GRFs and joint angles, identifying asymmetrical loading patterns in PD patients. Similarly, Slijepcevic et al. (2021) utilized LRP to highlight GRF features distinguishing hip, knee, and ankle pathologies from healthy gait. These studies demonstrated LRP’s ability to localize biomechanically critical phases of the gait cycle, such as mid-stance and push-off, which are often altered in neuromuscular disorders.

Attention mechanisms and Grad-CAM were adopted to provide temporal and spatial interpretability for recurrent and convolutional architectures (Apostolidis et al., 2023; Slijepcevic et al., 2023; Kim et al., 2024; Xiang et al., 2024). Xiang et al. (2024) applied an attention-based LSTM-MLP model to regress joint torques and contact forces, with attention weights pinpointing key temporal segments in healthy gait. Grad-CAM, used in three studies (Apostolidis et al., 2023; Slijepcevic et al., 2023; Kim et al., 2024), generated gradient-weighted activation maps to identify discriminative features in IMU and kinematic data. Apostolidis et al. (2023) applied high-activation regions in chronic stroke survivors to compensatory pelvic tilt strategies during stance phase.

##### 3.3.2.2 Interpretable statistical learning

SFLDA was employed by Yoon et al. (2023) as a transparent statistical method to classify gait patterns in CP. By enforcing sparsity constraints, SFLDA identified a minimal subset of discriminative joint angle features (e.g., hip flexion and knee abduction) that differentiated CP patients from controls. This approach provided clinically interpretable coefficients, enabling direct comparison with biomechanical literature on CP gait deviations.

##### 3.3.2.3 Comparison of different approaches

Neural network-based techniques enabled the moding of nonlinear, spatiotemporal relationships in high-dimensional input such as GRFs and IMU data. LRP and Grad-CAM revealed nonlinear interactions in CNNs, such as phase-dependent coupling between ankle dorsiflexion and GRF peaks in PD (Alharthi, 2024). In contract, SFLDA provided linear, population-level biomarkers for CP (Yoon et al., 2023).

Across studies, gait data were primarily collected using motion capture systems and wearable sensors, with ground reaction forces and joint angles among the most commonly analyzed features. Interpretability metrics, such as relevance scores and activation maps, were often validated against clinical assessments, including freezing of gait (FOG) severity in PD (Filtjens et al., 2021) and CP Gross Motor Function Classification System levels (GMFCS) (Slijepcevic et al., 2023). A key limitation was the lack of standardized frameworks for translating relevance scores into actionable clinical insights, particularly in studies with limited sample sizes (Xiang et al., 2024).

#### 3.3.3 Hybrid interpretability approaches

Three studies employed hybrid interpretability techniques to manage the complexity of gait analysis, combining attention mechanisms, graph-based modeling, and causal inference (Hou et al., 2022; Gu et al., 2024; Guo et al., 2024) (Table 5).

**TABLE 5:** Hybrid interpretability approaches in machine learning applied to gait analysis.

Studies	Population characteristic: number (group: age, males\females)	Gait analysis method	Machine learning algorithm	Explainable approach	Problem type	Data type	Interpretability-related metrics
Hou et al., 2022	10431 HP	Mocap	CNN	Squeeze-and-excitation mechanism, Part-based scoring	Classification	Images	Attention weights, Relevance Scores
Gu et al. (2024)	32 (17 CAI, 18–45 years, 6/11; 15 controls, 18–45 years, 5/10)	Mocap	GNN, ARL	Attention mechanism	Classification	Time-series joint coordinates	Attention weights
Guo et al. (2024)	273 (273 PD with FOG: 64.5 years, 188/85)	Mocap	Enhanced GCN	Causal inference, temporal and spatial localization	Classification	Video-based joint coordinates)	Attention weights, Relevance Scores, CES

HP, healthy participants; Mocap, Motion Capture; ARL, attention reinforcement learning, CAI, chronic ankle instability, CES, causal explanation subgraphs, GCN, graph convolutional network, GNN, graph neural networks, MAE, mean absolute error, PD, Parkinson’s Disease, FOG, freezing of gait.

##### 3.3.3.1 Attention-driven interpretability

Hou et al. (2022) and Gu et al. (2024) combined attention mechanisms with architectural innovations to enhance transparency. Hou et al. (2022) employed a Squeeze-and-Excitation Mechanism (channel-wise attention) and Part-based Scoring (spatial attention) in a CNN to classify gait patterns from mocap-derived images, identifying key anatomical regions (e.g., knee flexion) via attention weights. Gu et al. (2024) applied attention reinforcement learning (ARL) within a graph neural network (GNN) to analyze time-series joint coordinate data from individuals with chronic ankle instability (CAI), with attention weights highlighting asymmetrical ankle kinematics during stance.

##### 3.3.3.2 Causality-enhanced graph-based interpretability

Guo et al. (2024) introduced a causality-driven framework for classifying FOG in Parkinson’s disease. This model based on an Enhanced Graph Convolutional Network (GCN), the model integrated temporal-spatial graph convolutions (TSGCN) and multiple instance learning (MIL) to detect FOG episodes in video-based gait recording. A causal explanation framework quantified feature contributions, revealing that hip adduction and stride variability were causally linked to FOG onset. Performance metrics (accuracy = 92%, CES = 0.85) validated both predictive and explanatory power.

##### 3.3.3.3 Data and clinical relevance

The hybrid methods utilized diverse data types, including images (Hou et al., 2022), time-series joint coordinates (Gu et al., 2024), and video (Guo et al., 2024), to address population-specific challenges (e.g., CAI, PD-FOG). Attention mechanisms improved granularity for localized features, causal inference provided biomechanically plausible explanations for complex gait pathologies.

## 4 Discussion

This systematic review explores the application of XAI methods in gait analysis, providing insights into their prevalence, effectiveness, and limitations. Our comprehensive search across four databases identified 31 relevant studies, which we categorized based on their interpretability approaches. The findings highlight the widespread use of model-agnostic methods, particularly global and local interpretation techniques, and emphasize the role of XAI in analyzing gait patterns, especially in clinical populations. Notably, most studies focused on persons with neurological and musculoskeletal conditions, showcasing the potential of XAI to improve clinical decision-making and rehabilitation strategies.

### 4.1 Prevalence and variety of XAI approaches

This review underscores the versatility of model-agnostic interpretability techniques, which were employed in 16 studies (Dindorf et al., 2020; Davis et al., 2021; Kim et al., 2021; Kim et al., 2022; Rupprechter et al., 2021; Teufl et al., 2021; Kokkotis et al., 2022; Moon et al., 2022; Fan et al., 2023; Zheng et al., 2025; Aghababa and Andrysek, 2024; Hussain and Jany, 2024; Mulwa et al., 2024; Özateş et al., 2024; Trabassi et al., 2024; Wu et al., 2024). These techniques, such as SHAP, LIME, LRP, and Grad-CAM, were particularly favored due to their flexibility in application across various ML models. Global interpretation methods, such as SHAP (Lundberg and Lee, 2017), identified population-level biomarkers, such as reduced cadence in aging adults (Aziz et al., 2024; Bassan et al., 2024). In contrast, local interpretation techniques, such as Grad-CAM, enabled case-specific visualizations of influential gait features, offering clinicians a more intuitive understanding of model predictions (Rupprechter et al., 2021). Grad-CAM has been used to visualize phase-specific muscle activation patterns in sarcopenia, aligning with clinical gait assessments (Kim et al., 2022). Feature importance methods were extensively used to rank biomechanical variables, such as stride length and joint angles, bridging the gap between model predictions and biomechanical understanding (Dindorf et al., 2020; Teufl et al., 2021; Mulwa et al., 2024; Özateş et al., 2024). LRP and attention mechanisms are extensively used with CNN and LSTM models due to their ability to highlight relevant input features and improve interpretability in temporal data (Binder et al., 2016; Niu et al., 2021). This approach facilitated the identification of crucial gait features contributing to specific conditions, aiding both researchers and clinicians in interpreting ML-generated results effectively. However, only one included study evaluated the fidelity of local explanations, i.e., the extent to which the explanation accurately represented the model’s decision logic.

### 4.2 Pathological gait

A significant proportion of the reviewed studies concentrated on individuals with neurological and musculoskeletal conditions, including PD, stroke, sarcopenia, cerebral palsy, and musculoskeletal injuries (Filtjens et al., 2021; Kim et al., 2021; Kim et al., 2022; Slijepcevic et al., 2023; Yoon et al., 2023; Alharthi, 2024; Guo et al., 2024; Hussain and Jany, 2024; Mulwa et al., 2024). The complexity and heterogeneity of gait impairments in these populations necessitate advanced analytical methods, making XAI a valuable tool in clinical settings. For example, XAI methods have been instrumental in identifying gait features associated with FOG in PD (Filtjens et al., 2021; Guo et al., 2024) or compensatory mechanisms in stroke survivors (Apostolidis et al., 2023; Hussain and Jany, 2024). These insights have significant implications for targeted rehabilitation strategies and clinical decision-making, as interpretable ML models can offer transparent predictions regarding therapeutic outcomes and disease progression (Saraswat et al., 2022; Aziz et al., 2024). The emphasis on non-healthy populations also highlights the need for XAI techniques that can accommodate the variability inherent in pathological gait patterns (Slijepcevic et al., 2021).

### 4.3 Sensor modalities and data types

The reviewed studies utilized diverse sensor modalities, including Mocap, IMUs, EMG, and force plate, each with unique implications for interpretability and model accuracy. Mocap systems were widely used due to their precision in kinematic data collection, making them ideal for studies requiring detailed biomechanical indices. However, their reliance on controlled laboratory environments limits real-world applicability (Xiang et al., 2022b). IMUs captured dynamic movement patterns (e.g., stride variability in PD) (Wu et al., 2024), but their susceptibility to motion artifacts limited relevance score consistency (Creagh et al., 2021). Hybrid approaches, such as fusing IMU data with Mocap-derived kinematics (Zheng et al., 2025), may mitigate these issues. EMG sensors provided valuable muscle activation data, enhancing the interpretability of ML models. However, they require meticulous placement and calibration, introducing variability that can affect reproducibility (Karamanidis et al., 2004).

### 4.4 Machine learning models and the challenge of interpretability

Tree-based ensemble methods were among the most frequently used ML techniques due to their robustness in handling high-dimensional gait data and partial interpretability (Hussain and Jany, 2024). Support Vector Machine (SVM) also appeared frequently, benefiting from their simplicity and effectiveness in classification tasks (Dindorf et al., 2020). Despite their advantages, these models struggle with capturing intricate gait dynamics compared to more complex deep learning models. While deep learning models provide superior predictive power, their “black-box” nature hinders their adoption in clinical applications where transparency is paramount. As Rudin (2019) emphasized, reliance on *post hoc* explanations for black-box models in high-stakes decision-making may perpetuate poor practices and even cause harm, whereas inherently interpretable models offer a more transparent and trustworthy alternative. This perspective underscores the importance of balancing predictive performance with interpretability in gait-related applications, where clinical trust is critical. Nevertheless, integrating XAI methods into deep learning models remains essential to ensure that clinicians and researchers can interpret and rely on the predictions generated by advanced ML approaches. Deep learning-specific XAI techniques, such as LRP, Grad-CAM, and attention mechanisms, are particularly valuable in this context (Horst et al., 2019; Slijepcevic et al., 2023; Xiang et al., 2024).

Interpretability-related metrics are inherently heterogeneous across XAI methods and thus difficult to quantify in a standardized way. For example, feature attribution approaches (e.g., SHAP, LIME) are often assessed with fidelity or sensitivity measures, while saliency-based methods (e.g., Grad-CAM) are more commonly evaluated through visual plausibility or human judgment. This lack of cross-method comparability highlights the need for a structured framework that combines (i) method-specific fidelity metrics, (ii) user-centered evaluation of explanation usefulness, and (iii) transparent reporting of model accuracy thresholds.

### 4.5 Interplay between prediction and interpretability

The interpretability of ML models in gait analysis depends significantly on the quality and relevance of input data. Motion capture data, with its high fidelity, is preferred for biomechanical studies requiring precise kinematic analyses, whereas IMU data is often sufficient for broader classification tasks where portability is prioritized (Xiang et al., 2022b; Xiang et al., 2024). The effectiveness of XAI techniques also varies based on the prediction task. Classification tasks, such as distinguishing between healthy and pathological gait, frequently rely on feature importance methods like SHAP and LIME, which highlight key predictive features (Dindorf et al., 2020; Rupprechter et al., 2021). In contrast, regression tasks, such as estimating joint torques or stride length, require techniques that capture continuous relationships between input and output variables. While classification tasks dominate the literature due to their straightforward data labeling and model evaluation, regression tasks present a unique challenge for explainability. Emerging techniques, such as attention mechanisms (Xiang et al., 2024) and LRP (Horst et al., 2019), show promise in improving interpretability for these models by identifying influential input factors and revealing complex biomechanical relationships.

### 4.6 Limitations and future directions

Despite the advantages of XAI, challenges remain in achieving meaningful interpretability and enhancing trust and transparency, particularly in clinical settings. Ghassemi et al. (2021) argue that the potential of XAI may be overstated, warning against an overreliance on explainability as a strict requirement for clinical deployment. They liken it to human decision-making, where we often trust our judgments without fully understanding the underlying neural mechanisms. Similarly, while ML models—especially deep learning—can be highly complex and opaque, this does not necessarily prevent their effective use in healthcare. Different XAI methods pose challenges for standardization. For instance, SHAP values and relevance scores vary in their interpretation, making it difficult to establish consistent benchmarks. A key limitation identified is that most studies reported the type of XAI method applied without providing quantitative measures such as fidelity, consistency, or stability scores. Future research should complement qualitative explanations with standardized fidelity metrics to better evaluate how well XAI methods reflect underlying model behavior and to facilitate comparisons across studies. Standardizing XAI metrics such as fidelity and faithfulness is essential to ensure robustness, efficiency, and clinical applicability of XAI in gait biomechanics.

## 5 Conclusion

In summary, XAI holds promise for improving transparency and fostering clinical trust in gait-related machine learning. However, its effective translation requires refinement and validation. Based on our findings, we propose a practical roadmap: (i) developers should report not only model accuracy but also method-specific fidelity or consistency metrics to demonstrate explanation reliability; (ii) researchers should adopt standardized reporting practices that specify the XAI approach, dataset type, and evaluation criteria; and (iii) clinicians should critically appraise whether explanations are interpretable and actionable in their decision-making context, and participate in user-centered evaluations of XAI tools. Advancing along these lines will accelerate the clinical utility of XAI in gait analysis and rehabilitation.

## Data Availability

The original contributions presented in the study are included in the article/Supplementary Material, further inquiries can be directed to the corresponding author.

## References

[B1] Adadi A. Berrada M. (2018). Peeking inside the black-box: a survey on explainable artificial intelligence (XAI). IEEE access 6, 52138–52160. 10.1109/access.2018.2870052

[B2] Aeles J. Horst F. Lapuschkin S. Lacourpaille L. Hug F. (2021). Revealing the unique features of each individual’s muscle activation signatures. J. R. Soc. Interface 18, 20200770. 10.1098/rsif.2020.0770 33435843 PMC7879771

[B3] Aghababa M. P. Andrysek J. (2024). Exploration and demonstration of explainable machine learning models in prosthetic rehabilitation-based gait analysis. PLoS One 19, e0300447. 10.1371/journal.pone.0300447 38564508 PMC10987001

[B4] Albahri A. S. Duhaim A. M. Fadhel M. A. Alnoor A. Baqer N. S. Alzubaidi L. (2023). A systematic review of trustworthy and explainable artificial intelligence in healthcare: assessment of quality, bias risk, and data fusion. Inf. Fusion 96, 156–191. 10.1016/j.inffus.2023.03.008

[B5] Alharthi A. S. (2024). Interpretable machine learning comprehensive human gait deterioration analysis. Front. Neuroinform 18, 1451529. 10.3389/fninf.2024.1451529 39247901 PMC11377268

[B6] Apostolidis K. Kokkotis C. Karakasis E. Karampina E. Moustakidis S. Menychtas D. (2023). Innovative visualization approach for biomechanical time series in stroke diagnosis using explainable machine learning methods: a proof-of-concept study. Information 14, 559. 10.3390/info14100559

[B7] Aziz N. A. Manzoor A. Mazhar Qureshi M. D. Qureshi M. A. Rashwan W. (2024). Unveiling explainable AI in healthcare: current trends, challenges, and future directions. Preprint Repository Name [Preprint]. Available online at: https://persistent-url.

[B8] Bassan S. Amir G. Katz G. (2024). Local vs. global interpretability: a computational complexity perspective. ArXiv Prepr. 10.48550/arXiv.2406.02981

[B9] Binder A. Montavon G. Lapuschkin S. Müller K.-R. Samek W. (2016). “Layer-wise relevance propagation for neural networks with local renormalization layers,” in Artificial Neural Networks and Machine Learning–ICANN 2016: 25Th international conference on artificial neural networks, Barcelona, Spain, September 6-9, 2016, proceedings, part II 25 (Springer), 63–71.

[B10] Chen S. Lach J. Lo B. Yang G.-Z. (2016). Toward pervasive gait analysis with wearable sensors: a systematic review. IEEE J. Biomed. Health Inf. 20, 1521–1537. 10.1109/jbhi.2016.2608720 28113185

[B11] Cirstea C. M. (2020). Gait rehabilitation after stroke: should we re-evaluate our practice? Stroke 51, 2892–2894. 10.1161/strokeaha.120.032041 32912098

[B12] Creagh A. P. Lipsmeier F. Lindemann M. Vos M.De (2021). Interpretable deep learning for the remote characterisation of ambulation in multiple sclerosis using smartphones. Sci. Rep. 11, 14301. 10.1038/s41598-021-92776-x 34253769 PMC8275610

[B13] Davis J. R. C. Knight S. P. Donoghue O. A. Hernández B. Rizzo R. Kenny R. A. (2021). Comparison of gait speed reserve, usual gait speed, and maximum gait speed of adults aged 50+ in Ireland using explainable machine learning. Front. Netw. Physiology 1, 754477. 10.3389/fnetp.2021.754477 36925580 PMC10013005

[B14] Dindorf C. Teufl W. Taetz B. Bleser G. Fröhlich M. (2020). Interpretability of input representations for gait classification in patients after total hip arthroplasty. Sensors 20, 4385. 10.3390/s20164385 32781583 PMC7471970

[B15] Dou H. Zhang P. Su W. Yu Y. Lin Y. Li X. (2023). “Gaitgci: generative counterfactual intervention for gait recognition,” in Proceedings of the IEEE/CVF conference on computer vision and pattern recognition, 5578–5588.

[B16] Downs S. H. Black N. (1998). The feasibility of creating a checklist for the assessment of the methodological quality both of randomised and non-randomised studies of health care interventions. J. Epidemiol. Community Health 52, 377–384. 10.1136/jech.52.6.377 9764259 PMC1756728

[B17] Faerman M. V. Cole C. Van Ooteghem K. Cornish B. F. Howe E. E. Siu V. (2025). Motor, affective, cognitive, and perceptual symptom changes over time in individuals with Parkinson’s disease who develop freezing of gait. J. Neurol. 272, 321–19. 10.1007/s00415-025-13034-y 40198411

[B18] Fan S. Ye J. Xu Q. Peng R. Hu B. Pei Z. (2023). Digital health technology combining wearable gait sensors and machine learning improve the accuracy in prediction of frailty. Front. Public Health 11, 1169083. 10.3389/fpubh.2023.1169083 37546315 PMC10402732

[B19] Ferber R. Osis S. T. Hicks J. L. Delp S. L. (2016). Gait biomechanics in the era of data science. J. Biomech. 49, 3759–3761. 10.1016/j.jbiomech.2016.10.033 27814971 PMC5407492

[B20] Filtjens B. Ginis P. Nieuwboer A. Afzal M. R. Spildooren J. Vanrumste B. (2021). Modelling and identification of characteristic kinematic features preceding freezing of gait with convolutional neural networks and layer-wise relevance propagation. BMC Med. Inf. Decis. Mak. 21, 341–11. 10.1186/s12911-021-01699-0 34876110 PMC8650332

[B21] Frasca M. La Torre D. Pravettoni G. Cutica I. (2024). Explainable and interpretable artificial intelligence in medicine: a systematic bibliometric review. Discov. Artif. Intell. 4, 15. 10.1007/s44163-024-00114-7

[B22] Gao Z. Xiang L. Fekete G. Baker J. S. Mao Z. Gu Y. (2023). A data-driven approach for fatigue detection during running using pedobarographic measurements. Appl. Bionics Biomech. 2023, 1–11. 10.1155/2023/7022513 37794856 PMC10547577

[B23] Ghassemi M. Oakden-Rayner L. Beam A. L. (2021). The false hope of current approaches to explainable artificial intelligence in health care. Lancet Digit. Health 3, e745–e750. 10.1016/s2589-7500(21)00208-9 34711379

[B24] Gu H. Yen S.-C. Folmar E. Chou C.-A. (2024). GaitNet+ ARL: a deep learning algorithm for interpretable gait analysis of chronic ankle instability. IEEE J. Biomed. Health Inf. 28, 3918–3927. 10.1109/jbhi.2024.3383588 38557612

[B25] Guo R. Xie Z. Zhang C. Qian X. (2024). Causality-enhanced multiple instance learning with graph convolutional networks for parkinsonian freezing-of-gait assessment. IEEE Trans. Image Process. 33, 3991–4001. 10.1109/tip.2024.3416052 38913508

[B26] Halilaj E. Rajagopal A. Fiterau M. Hicks J. L. Hastie T. J. Delp S. L. (2018). Machine learning in human movement biomechanics: best practices, common pitfalls, and new opportunities. J. Biomech. 81, 1–11. 10.1016/j.jbiomech.2018.09.009 30279002 PMC6879187

[B27] Harris E. J. Khoo I.-H. Demircan E. (2022). A survey of human gait-based artificial intelligence applications. Front. Robot. AI 8, 749274. 10.3389/frobt.2021.749274 35047564 PMC8762057

[B28] Horst F. Lapuschkin S. Samek W. Müller K.-R. Schöllhorn W. I. (2019). Explaining the unique nature of individual gait patterns with deep learning. Sci. Rep. 9, 2391. 10.1038/s41598-019-38748-8 30787319 PMC6382912

[B29] Horst F. Slijepcevic D. Zeppelzauer M. Raberger A.-M. Lapuschkin S. Samek W. (2020). Explaining automated gender classification of human gait. Gait Posture 81, 159–160. 10.1016/j.gaitpost.2020.07.114 31862670

[B30] Hou S. Liu X. Cao C. Huang Y. (2022). Gait quality aware network: toward the interpretability of silhouette-based gait recognition. IEEE Trans. Neural Netw. Learn Syst. 34, 8978–8988. 10.1109/tnnls.2022.3154723 35294358

[B31] Hussain I. Jany R. (2024). Interpreting stroke-impaired electromyography patterns through explainable artificial intelligence. Sensors 24, 1392. 10.3390/s24051392 38474928 PMC10935041

[B32] Karamanidis K. Arampatzis A. Brüggemann G.-P. (2004). Reproducibility of electromyography and ground reaction force during various running techniques. Gait Posture 19, 115–123. 10.1016/s0966-6362(03)00040-7 15013499

[B33] Kessler S. E. Rainbow M. J. Lichtwark G. A. Cresswell A. G. D’Andrea S. E. Konow N. (2019). A direct comparison of biplanar videoradiography and optical motion capture for foot and ankle kinematics. Front. Bioeng. Biotechnol. 7, 199. 10.3389/fbioe.2019.00199 31508415 PMC6716496

[B34] Kim J.-K. Bae M.-N. Lee K. B. Hong S. G. (2021). Identification of patients with sarcopenia using gait parameters based on inertial sensors. Sensors 21, 1786. 10.3390/s21051786 33806525 PMC7961754

[B35] Kim J.-K. Bae M.-N. Lee K. Kim J.-C. Hong S. G. (2022). Explainable artificial intelligence and wearable sensor-based gait analysis to identify patients with osteopenia and sarcopenia in daily life. Biosens. (Basel) 12, 167. 10.3390/bios12030167 35323437 PMC8946270

[B36] Kim Y.-G. Kim S. Park J. H. Yang S. Jang M. Yun Y. J. (2024). Explainable deep-learning-based gait analysis of hip–knee cyclogram for the prediction of adolescent idiopathic scoliosis progression. Sensors 24, 4504. 10.3390/s24144504 39065902 PMC11280687

[B37] Kokkotis C. Moustakidis S. Tsatalas T. Ntakolia C. Chalatsis G. Konstadakos S. (2022). Leveraging explainable machine learning to identify gait biomechanical parameters associated with anterior cruciate ligament injury. Sci. Rep. 12, 6647. 10.1038/s41598-022-10666-2 35459787 PMC9026057

[B38] Loh H. W. Ooi C. P. Seoni S. Barua P. D. Molinari F. Acharya U. R. (2022). Application of explainable artificial intelligence for healthcare: a systematic review of the last decade (2011–2022). Comput. Methods Programs Biomed. 226, 107161. 10.1016/j.cmpb.2022.107161 36228495

[B39] Lundberg S. M. Lee S.-I. (2017). “A unified approach to interpreting model predictions,” in Proceedings of the 31st Conference on Neural Information Processing Systems (NIPS 2017) 30.

[B40] Martínez-Pascual D. Catalán J. M. Blanco-Ivorra A. Sanchís M. Arán-Ais F. García-Aracil N. (2024). Gait activity classification with convolutional neural network using lower limb angle measurement from inertial sensors. IEEE Sens. J. 24, 21479–21489. 10.1109/JSEN.2024.3400296

[B41] Mekni A. Narayan J. Gritli H. (2025a). Multi-class classification of gait cycle phases using machine learning: a comprehensive study using two training methods. Netw. Model. Analysis Health Inf. Bioinforma. 14, 30. 10.1007/s13721-025-00522-4

[B42] Mekni A. Narayan J. Gritli H. (2025b). Quinary classification of human gait phases using machine learning: investigating the potential of different training methods and scaling techniques. Big Data Cognitive Comput. 9, 89. 10.3390/bdcc9040089

[B43] Minh D. Wang H. X. Li Y. F. Nguyen T. N. (2022). Explainable artificial intelligence: a comprehensive review. Artif. Intell. Rev. 55, 3503–3568. 10.1007/s10462-021-10088-y

[B44] Moher D. Liberati A. Tetzlaff J. Altman D. G. Group T. P. (2009). Preferred reporting items for systematic reviews and meta-analyses: the PRISMA statement. PLoS Med. 6, e1000097. 10.1371/journal.pmed.1000097 19621072 PMC2707599

[B45] Moon J. Shin Y.-M. Park J.-D. Minaya N. H. Shin W.-Y. Choi S.-I. (2022). Explainable gait recognition with prototyping encoder–decoder. PLoS One 17, e0264783. 10.1371/journal.pone.0264783 35275965 PMC8916664

[B46] Mulwa M. M. Mwangi R. W. Mindila A. (2024). GMM‐LIME explainable machine learning model for interpreting sensor‐based human gait. Eng. Rep. 6, e12864. 10.1002/eng2.12864

[B47] Niu Z. Zhong G. Yu H. (2021). A review on the attention mechanism of deep learning. Neurocomputing 452, 48–62. 10.1016/j.neucom.2021.03.091

[B48] Özateş M. E. Yaman A. Salami F. Campos S. Wolf S. I. Schneider U. (2024). Identification and interpretation of gait analysis features and foot conditions by explainable AI. Sci. Rep. 14, 5998. 10.1038/s41598-024-56656-4 38472287 PMC10933258

[B49] Phinyomark A. Petri G. Ibáñez-Marcelo E. Osis S. T. Ferber R. (2018). Analysis of big data in gait biomechanics: current trends and future directions. J. Med. Biol. Eng. 38, 244–260. 10.1007/s40846-017-0297-2 29670502 PMC5897457

[B50] Ribeiro M. T. Singh S. Guestrin C. (2016). “Why should i trust you? Explaining the predictions of any classifier,” in Proceedings of the 22nd ACM SIGKDD international conference on knowledge discovery and data mining, 1135–1144.

[B51] Rudin C. (2019). Stop explaining Black box machine learning models for high stakes decisions and use interpretable models instead. Nat. Mach. Intell. 1, 206–215. 10.1038/s42256-019-0048-x 35603010 PMC9122117

[B52] Rupprechter S. Morinan G. Peng Y. Foltynie T. Sibley K. Weil R. S. (2021). A clinically interpretable computer-vision based method for quantifying gait in parkinson’s disease. Sensors 21, 5437. 10.3390/s21165437 34450879 PMC8399017

[B53] Salahuddin Z. Woodruff H. C. Chatterjee A. Lambin P. (2022). Transparency of deep neural networks for medical image analysis: a review of interpretability methods. Comput. Biol. Med. 140, 105111. 10.1016/j.compbiomed.2021.105111 34891095

[B54] Saraswat D. Bhattacharya P. Verma A. Prasad V. K. Tanwar S. Sharma G. (2022). Explainable AI for healthcare 5.0: opportunities and challenges. IEEe Access 10, 84486–84517. 10.1109/access.2022.3197671

[B55] Selvaraju R. R. Cogswell M. Das A. Vedantam R. Parikh D. Batra D. (2017). “Grad-cam: visual explanations from deep networks *via* gradient-based localization,” in Proceedings of the IEEE international conference on computer vision, 618–626.

[B56] Slijepcevic D. Horst F. Lapuschkin S. Horsak B. Raberger A.-M. Kranzl A. (2021). Explaining machine learning models for clinical gait analysis. ACM Trans. Comput. Healthc. Heal. 3, 1–27. 10.1145/3474121

[B57] Slijepcevic D. Zeppelzauer M. Unglaube F. Kranzl A. Breiteneder C. Horsak B. (2023). Explainable machine learning in human gait analysis: a study on children with cerebral palsy. IEEE Access 11, 65906–65923. 10.1109/access.2023.3289986

[B58] Teoh Y. X. Othmani A. Goh S. L. Usman J. Lai K. W. (2024). Deciphering knee osteoarthritis diagnostic features with explainable artificial intelligence: a systematic review. IEEE Access 12, 109080–109108. 10.1109/access.2024.3439096

[B59] Teufl W. Taetz B. Miezal M. Dindorf C. Fröhlich M. Trinler U. (2021). Automated detection and explainability of pathological gait patterns using a one-class support vector machine trained on inertial measurement unit based gait data. Clin. Biomech. 89, 105452. 10.1016/j.clinbiomech.2021.105452 34481198

[B60] Tjoa E. Guan C. (2020). A survey on explainable artificial intelligence (Xai): toward medical xai. IEEE Trans. Neural Netw. Learn Syst. 32, 4793–4813. 10.1109/tnnls.2020.3027314 33079674

[B61] Trabassi D. Castiglia S. F. Bini F. Marinozzi F. Ajoudani A. Lorenzini M. (2024). Optimizing rare disease gait classification through data balancing and generative AI: insights from hereditary cerebellar ataxia. Sensors 24, 3613. 10.3390/s24113613 38894404 PMC11175240

[B62] Uhlrich S. D. Falisse A. Kidziński Ł. Muccini J. Ko M. Chaudhari A. S. (2023). OpenCap: human movement dynamics from smartphone videos. PLoS Comput. Biol. 19, e1011462. 10.1371/journal.pcbi.1011462 37856442 PMC10586693

[B63] Vilone G. Longo L. (2021). Notions of explainability and evaluation approaches for explainable artificial intelligence. Inf. Fusion 76, 89–106. 10.1016/j.inffus.2021.05.009

[B64] Wade L. Needham L. McGuigan P. Bilzon J. (2022). Applications and limitations of current markerless motion capture methods for clinical gait biomechanics. PeerJ 10, e12995. 10.7717/peerj.12995 35237469 PMC8884063

[B65] Winter D. A. (2009). Biomechanics and motor control of human movement. Hoboken, NJ: John Wiley & Sons.

[B66] Wu X. Ma L. Wei P. Shan Y. Chan P. Wang K. (2024). Wearable sensor devices can automatically identify the ON-OFF status of patients with Parkinson’s disease through an interpretable machine learning model. Front. Neurol. 15, 1387477. 10.3389/fneur.2024.1387477 38751881 PMC11094303

[B67] Xiang L. Gu Y. Mei Q. Wang A. Shim V. Fernandez J. (2022a). Automatic classification of barefoot and shod populations based on the foot metrics and plantar pressure patterns. Front. Bioeng. Biotechnol. 10, 843204. 10.3389/fbioe.2022.843204 35402419 PMC8984198

[B68] Xiang L. Wang A. Gu Y. Zhao L. Shim V. Fernandez J. (2022b). Recent machine learning progress in lower limb running biomechanics with wearable technology: a systematic review. Front. Neurorobot 16, 913052. 10.3389/fnbot.2022.913052 35721274 PMC9201717

[B69] Xiang L. Gu Y. Wang A. Shim V. Gao Z. Fernandez J. (2023). Foot pronation prediction with inertial sensors during running: a preliminary application of data-driven approaches. J. Hum. Kinet. 88, 29–40. 10.5114/jhk/163059 37559759 PMC10407326

[B70] Xiang L. Gu Y. Gao Z. Yu P. Shim V. Wang A. (2024). Integrating an LSTM framework for predicting ankle joint biomechanics during gait using inertial sensors. Comput. Biol. Med. 170, 108016. 10.1016/j.compbiomed.2024.108016 38277923

[B75] Xiang L. Gu Y. Deng K. Gao Z. Shim V. Wang A. (2025). Integrating personalized shape prediction, biomechanical modeling, and wearables for bone stress prediction in runners. Npj Digit. Med. 8, 276. 10.1038/s41746-025-01677-0 40360731 PMC12075602

[B71] Yang G. Ye Q. Xia J. (2022). Unbox the black-box for the medical explainable AI *via* multi-modal and multi-centre data fusion: a mini-review, two showcases and beyond. Inf. Fusion 77, 29–52. 10.1016/j.inffus.2021.07.016 34980946 PMC8459787

[B72] Yoon C. Jeon Y. Choi H. Kwon S.-S. Ahn J. (2023). Interpretable classification for multivariate gait analysis of cerebral palsy. Biomed. Eng. Online 22, 109. 10.1186/s12938-023-01168-x 37993868 PMC10664661

[B73] Zheng X. Otten B. Reneman M. F. Lamoth C. J. (2025). Explaining deep learning models for age-related gait classification based on acceleration time series. Comput. Biol. Med. 184, 109338. 10.1016/j.compbiomed.2024.109338 39536383

