# Supplementary material for: Explainable artificial intelligence for gait analysis: advances, pitfalls, and challenges - a systematic review
Source: Front Bioeng Biotechnol. 2025 Oct 30;13:1671344. doi: 10.3389/fbioe.2025.1671344 (PMC12611957; doi:10.3389/fbioe.2025.1671344)
Supplement: Supplementary file 1 [file Table1.docx]

Supplementary Material

# Supplementary Table

**Table S1.** Quality assessment scoring of 31 included studies.

| **Study** | **Q1** | **Q2** | **Q3** | **Q4** | **Q5** | **Q6** | **Q7** | **Q8** | **Q9** | **Q10** | **Q11** | **Q12** | **Total** | **%** |
| --- | --- | --- | --- | --- | --- | --- | --- | --- | --- | --- | --- | --- | --- | --- |
| Kim et al., 2022 | 2 | 2 | 2 | 2 | 1 | 2 | 2 | 2 | 2 | 1.5 | 1.5 | 1.5 | 21.5 | 89.60% |
| Rupprechter et al., 2021 | 2 | 2 | 2 | 2 | 1 | 1.5 | 2 | 2 | 2 | 2 | 1.5 | 1 | 21 | 87.50% |
| Kim et al., 2021 | 2 | 2 | 2 | 2 | 1 | 1.5 | 2 | 2 | 2 | 1.5 | 1.5 | 1.5 | 21.5 | 89.60% |
| Aghababa et al., 2024 | 2 | 2 | 2 | 2 | 1 | 1.5 | 2 | 2 | 2 | 1.5 | 1.5 | 1 | 20.5 | 85.40% |
| Kokkotis et al., 2022 | 2 | 2 | 2 | 2 | 1 | 1.5 | 2 | 2 | 2 | 1.5 | 1.5 | 1.5 | 21.5 | 89.60% |
| Wu et al., 2024 | 2 | 2 | 2 | 2 | 1 | 1.5 | 2 | 2 | 2 | 1.5 | 1.5 | 1.5 | 21.5 | 89.60% |
| Davis et al., 2021 | 2 | 2 | 2 | 1 | 1.5 | 1.5 | 2 | 1.5 | 2 | 1.5 | 2 | 1 | 20 | 83.30% |
| Fan et al., 2023 | 2 | 2 | 2 | 2 | 1.5 | 1.5 | 2 | 2 | 2 | 2 | 1.5 | 1.5 | 22 | 91.70% |
| Hussain et al., 2024 | 2 | 2 | 2 | 2 | 1 | 2 | 2 | 2 | 2 | 1.5 | 1.5 | 1.5 | 21.5 | 89.60% |
| Kim et al., 2024 | 2 | 2 | 2 | 2 | 1 | 2 | 2 | 2 | 2 | 1.5 | 1.5 | 1.5 | 21.5 | 89.60% |
| Apostolidis et al., 2023 | 2 | 2 | 2 | 2 | 1 | 1.5 | 2 | 2 | 2 | 1.5 | 1.5 | 1.5 | 21.5 | 89.60% |
| Mulwa et al., 2024 | 2 | 2 | 2 | 2 | 1 | 2 | 2 | 2 | 2 | 1.5 | 1.5 | 1.5 | 21.5 | 89.60% |
| Özateş et al., 2024 | 2 | 2 | 2 | 2 | 1 | 2 | 2 | 2 | 2 | 1.5 | 1.5 | 1.5 | 21.5 | 89.60% |
| Dindorf et al., 2020 | 2 | 2 | 2 | 2 | 1 | 1.5 | 2 | 2 | 2 | 1.5 | 1.5 | 1 | 20.5 | 85.40% |
| Moon et al., 2022 | 2 | 2 | 2 | 2 | 1 | 1.5 | 2 | 2 | 2 | 1.5 | 1.5 | 1.5 | 21 | 87.50% |
| Slijepcevic et al., 2023 | 2 | 2 | 2 | 2 | 1 | 2 | 2 | 2 | 2 | 1.5 | 1.5 | 1.5 | 21.5 | 89.60% |
| Teufl et al., 2021 | 2 | 2 | 2 | 2 | 1.5 | 1.5 | 2 | 2 | 2 | 2 | 1.5 | 1 | 21.5 | 89.60% |
| Horst et al., 2019 | 2 | 2 | 2 | 2 | 1 | 2 | 2 | 2 | 2 | 1.5 | 1.5 | 1.5 | 21.5 | 89.60% |
| Alharthi et al., 2023 | 2 | 2 | 2 | 2 | 1 | 1.5 | 2 | 2 | 2 | 1.5 | 1.5 | 1.5 | 21.5 | 89.60% |
| Slijepcevic et al., 2021 | 2 | 2 | 2 | 2 | 1 | 2 | 2 | 2 | 2 | 1.5 | 1.5 | 1.5 | 21.5 | 89.60% |
| Horst et al., 2020 | 2 | 2 | 2 | 2 | 1 | 1.5 | 2 | 2 | 1.5 | 2 | 1 | 1.5 | 20.5 | 85.40% |
| Filtjens et al., 2021 | 2 | 2 | 2 | 2 | 1 | 1.5 | 2 | 2 | 2 | 1.5 | 1.5 | 1.5 | 21.5 | 89.60% |
| Aeles et al., 2021 | 2 | 2 | 2 | 2 | 1 | 1.5 | 2 | 2 | 2 | 1.5 | 1.5 | 1.5 | 21.5 | 89.60% |
| Creagh et al., 2021 | 2 | 2 | 2 | 2 | 1 | 1.5 | 2 | 2 | 2 | 1.5 | 1.5 | 1.5 | 21.5 | 89.60% |
| Xiang et al., 2024 | 2 | 2 | 2 | 2 | 1 | 1.5 | 2 | 2 | 2 | 1.5 | 1.5 | 1.5 | 21.5 | 89.60% |
| Hou et al., 2023 | 2 | 2 | 2 | 2 | 1 | 1.5 | 2 | 2 | 2 | 1.5 | 1.5 | 1 | 20.5 | 85.40% |
| Gu et al., 2024 | 2 | 2 | 2 | 2 | 1 | 2 | 2 | 2 | 2 | 1.5 | 1.5 | 1.5 | 21.5 | 89.60% |
| Yoon et al., 2023 | 2 | 2 | 2 | 2 | 1 | 2 | 2 | 2 | 2 | 1.5 | 1.5 | 1.5 | 21.5 | 89.60% |
| Zheng et al., 2025 | 2 | 2 | 2 | 2 | 1 | 1.5 | 2 | 2 | 2 | 1.5 | 1.5 | 1.5 | 21 | 87.50% |
| Guo et al., 2024 | 2 | 2 | 2 | 2 | 1.5 | 1.5 | 2 | 2 | 1.5 | 1.5 | 1.5 | 1.5 | 21 | 87.50% |
| Trabassi et al., 2024 | 2 | 2 | 2 | 2 | 1 | 1.5 | 2 | 2 | 2 | 2 | 1.5 | 1.5 | 21.5 | 89.6% |
| **Average** | **2** | **2** | **2** | **1.97** | **1.10** | **1.66** | **2** | **1.98** | **1.97** | **1.58** | **1.5** | **1.4** | **21.27** | **88.65%** |
